# Mechanobiology of the blood-brain barrier during development, disease and ageing

**DOI:** 10.1038/s41467-025-61888-7

**Published:** 2025-08-06

**Authors:** Simon Konig, Vignesh Jayarajan, Selina Wray, Roger Kamm, Emad Moeendarbary

**Affiliations:** 1 Department of Mechanical Engineering, University College London, London, UK; 2 UCL Queen Square Institute of Neurology, University College London, London, UK; 3 Department of Biological Engineering, Massachusetts Institute of Technology, Cambridge, MA USA; 4 Department of Mechanical Engineering, Massachusetts Institute of Technology, Cambridge, MA USA; 5 BioRecode Ltd, London, England

**Keywords:** Blood-brain barrier, Engineering

## Abstract

The blood-brain barrier (BBB) preserves brain health through selective permeability, and its disruption is a hallmark of many neurological disorders. Mechanical stimuli such as shear stress and cyclic strain are increasingly recognised to influence BBB integrity and function, while alterations in tissue stiffness and extracellular matrix composition contribute to its breakdown during ageing and disease. Despite its importance, BBB mechanobiology remains underexplored. Here we highlight the central role of mechanics in BBB development, pathology, and ageing, identify key knowledge gaps, and argue that combining innovative BBB model systems with mechanical probing techniques could transform therapeutic strategies targeting brain vascular dysfunction.

## Abstract (web summary)

The mechanobiology of blood brain barrier (BBB) remains underexplored. In this perspective, the authors highlight the emerging importance of mechanical forces in shaping BBB function across development, disease, and ageing. They also argue that integrating biomechanics into BBB research is important for advancing future therapies.

## Introduction

A healthy blood–brain barrier (BBB) is essential for maintaining the delicate brain microenvironment and ensures cerebral homoeostasis. Its key function is to allow the selective bidirectional transport of molecules crucial for supporting critical brain functions and blocking the entry of toxins, pathogens, and other blood-derived products^1^. BBB dysfunction associated with increased BBB permeability has been observed as a consequence of ageing as well as in various neurological conditions, including hypoxic and ischaemic insults, epilepsy, brain tumours, neurodegenerative disorders (such as Parkinson’s and Alzheimer’s disease (AD)), and psychiatric conditions like depression, schizophrenia, and autism spectrum disorder^1–8^. During BBB dysfunction, emerging evidence suggests that BBB mechanics are altered, which may not be merely incidental but could play an active role in facilitating BBB breakdown. These mechanical changes are a natural part of development and ageing, but are also prominent features of various neuropathologies. While the broader field of mechanobiology has revealed that mechanics impact cell behaviour and tissue morphogenesis, the mechanobiology of the BBB remains an underexplored frontier. With the growing prevalence of neurodegenerative diseases, the BBB’s essential role in preserving neural homoeostasis and its sensitivity to mechanical disturbances demand thorough investigation. In this perspective, we provide comprehensive evidence highlighting the pivotal role of mechanics in normal BBB function while also exploring how mechanical forces and properties influence BBB development, ageing, and disease and what therapeutic opportunities could emerge from these insights.

## The blood-brain barrier: An overview

**Fig. 1:**
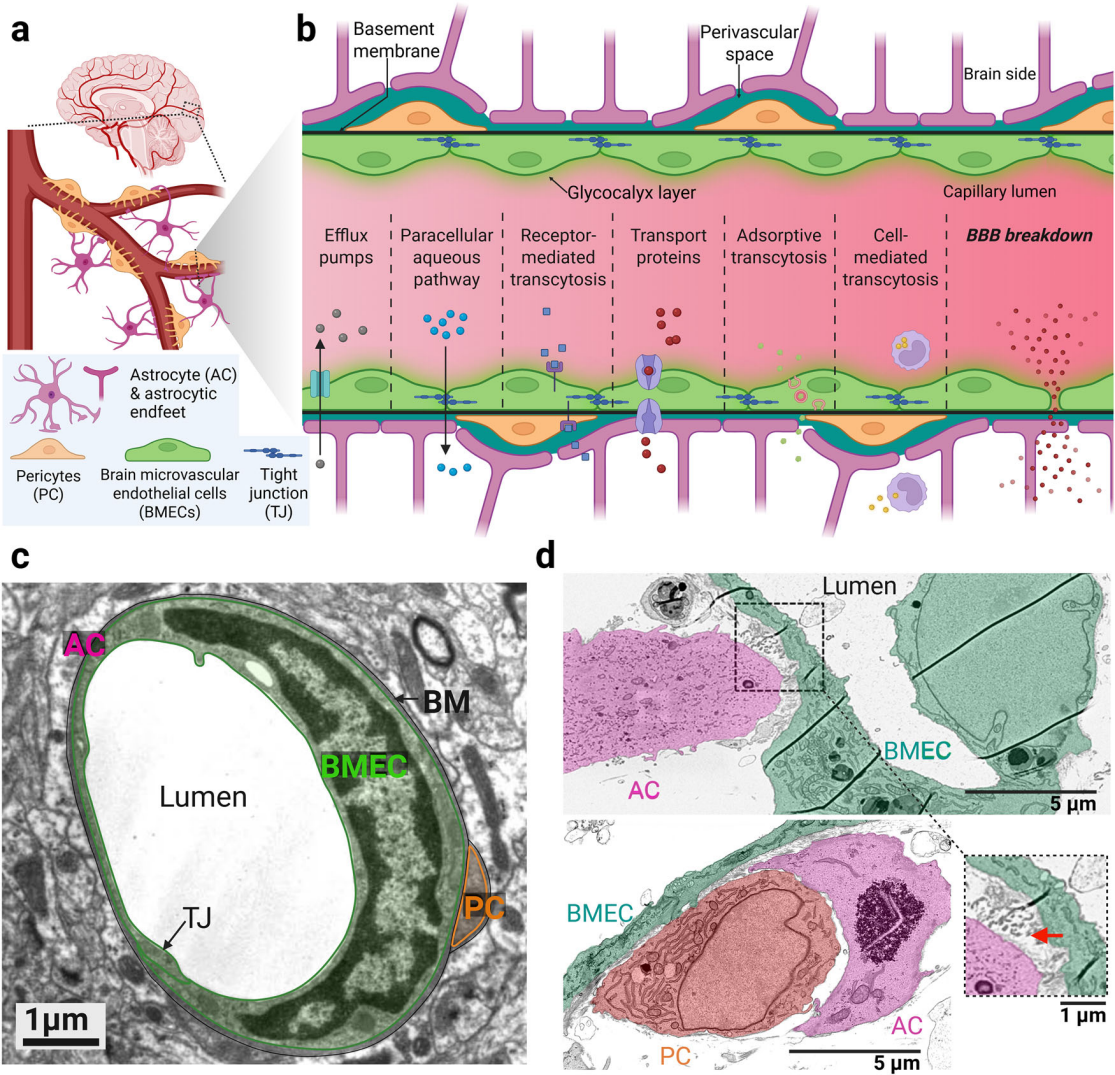
BBB structure and function. **a** Schematic representation of the BBB. The BBB is located at the brain capillaries and consists of brain microvascular endothelial cells (BMECs), pericytes (PC), and astrocytes (AC). **b** The BMECs are tightly linked by tight junction (TJ) proteins, thereby limiting non-aqueous paracellular diffusion and facilitating the entry of molecules by different transcellular pathways. During BBB breakdown, the TJ proteins are disrupted, allowing the free entry of blood-derived products into the brain. Created in BioRender. Konig, S. (2025) https://BioRender.com/eqcbpzi. **c** Cross-sectional scanning electron microscopy (SEM) image of a capillary of a mouse brain, showing a BMEC forming a TJ with itself. PCs and astrocytic end-feet align closely to the BMEC, which is surrounded by the basement membrane (BM). Adapted from Wolburg H, Noell S, Wolburg-Buchholz K, Mack A, Fallier-Becker P, Neuroscientist, pp. 180–193. Copyright © 2009 by Copyright Holder. Reprinted by Permission of Sage Publications^177^. **d** Corresponding SEM image of histological sections collected from a recently developed in vitro BBB model showing similar cellular interactions as in vivo, adapted from ref. ^178^. Stromal cells and the BMEC interact actively (magnified image in the inset), as witnessed by the increased vesicle density at the contact point between the astrocytic end-feet and the BMEC (red arrow).

Structurally, the BBB pertains primarily to the systemic capillaries, which are the smallest vessels of the cerebrovascular network (diameter ~3–7 μm, wall thickness ~0.1 μm) and the main location for blood–brain exchange^9,10^ (Fig. 1b).

Three main cell types, including brain microvascular endothelial cells (BMECs), pericytes, and astrocytes^9^, are primarily responsible for BBB function (Fig. 1a, b). These cells are in turn surrounded by specific extracellular matrix (ECM) components such as a vascular basement membrane and an intraluminal glycocalyx layer as well as a perivascular space that constitutes the glymphatic circulation and is believed to be a critical pathway for the clearance of substances such as amyloid beta from the brain^9,11^.

BMECs are generally considered to be the core constituting cells of the BBB because of their selective barrier properties^12^. Being located on the intraluminal side of the capillaries, they form a tight barrier by expressing complex tight junctions composed of proteins, such as claudin 5 (CLDN5), occludin, and the zonula occludens protein 1 (ZO-1), which limit paracellular diffusion and require the majority of large molecule transport to occur through the transcellular pathway^12,13^ (Fig. 1b). Here, various membrane transporters shuttle molecules passively or actively between the blood and the brain parenchyma^14^. Since both the tight junctions and the membrane transporters can undergo rapid modulation and regulation, BMECs allow the brain to adapt quickly to changing energy demands and efficiently secrete waste products^14^. Besides their specific expression profiles, BMECs also differ from peripheral endothelial cells by displaying a higher expression of mitochondrial content, a lack of fenestration, and a low degree of pinocytosis, thereby further complementing the effectiveness and selectivity of this endothelial barrier^15,16^.

The second type of BBB-associated cells are pericytes, which are embedded in the basement membrane and wrap around BMECs^17^ (Fig. 1c). They exhibit the highest capillary coverage in neural tissue, covering up to 37% of the extraluminal side of BMECs^18^. Throughout development and adulthood, pericytes are key players in BBB formation and regulation by actively communicating with BMECs^19^. Through paracrine and juxtacrine signalling, including “peg-and-socket” contacts, pericytes can induce the expression of BBB-specific genes in BMECs and regulate the number of tight junctions in them^19^. Importantly, pericyte-deficient animal models have shown regional differences in BBB permeability upon pericyte loss, possibly implicating a heterogenous distribution of pericytes^20^. Pericytes are also involved in other functions such as the clearance of toxic substances and neurovascular coupling, whereby they contract to alter capillary blood flow depending on the changing energy demands of the corresponding brain region^21–23^.

Astrocytes, the third type of BBB-associated cells, form astrocytic end-feet extensively covering the extraluminal side of BMECs and pericytes^18^ (Fig. 1c, d). They play a central role in mediating a sufficient supply of nutrients, such as glucose and oxygen, to regions of the brain with high synaptic activity and energy demands^24,25^. Hereby, astrocytes regulate smooth muscle cells in arterioles to induce vasodilation^25^. Additionally, they influence the expression and localisation of transporters and enzyme systems in BMECs^26^. They are also reportedly capable of altering the invasion of circulating cells, including immune and cancer cells, across the BBB. Hereby, astrocytes secrete chemokines and inflammatory cytokines that act on BMECs and change the expression of tight-junction proteins as well as cell adhesion molecules on BMECs, thereby promoting adhesion and transmigration^27–30^. It was also shown recently that astrocytes can promote the chemotaxis and chemokinesis of cancer cells directly without affecting vascular permeability^31^.

The basement membrane is a 20- to 200-nm-thick proteoglycan-rich matrix made of a network formed primarily by collagen IV and laminin isoforms held together by nidogen and perlecan, as well as other proteoglycans and enzymes^32,33^. While all three cell types form direct cell–cell junctions via proteins such as connexins and cadherins, all the cells are also tightly anchored within this abluminal basement membrane^34^. Besides its role in anchoring BBB-associated cells, the basement membrane also modulates various signalling mechanisms that are important for BBB function and maintenance. It also constitutes an important barrier limiting the entry or exit of tumour and immune cells^35^.

## Blood-brain barrier mechanobiology

Cells are mechanosensitive. They actively sense and respond to physical cues from their local cellular microenvironment^36^. Mechanotransduction, the process whereby mechanical signals trigger intracellular or biochemical signalling cascades, has been shown to alter the function and behaviour of various cell types within different pathophysiological processes^37,38^. A large body of evidence supports the importance of mechanics within the peripheral vasculature^39–41^. Generally, vascular permeability, tone, and inflammatory states are all tightly linked by regulated mechanobiological pathways^42–44^. Impaired mechanotransduction due to stiffened blood vessels and impaired flow patterns is thought to promote the development of cardiovascular diseases such as atherosclerosis^45,46^. Mechanics also likely plays an essential role at the BBB. However, given its unique architecture and barrier-forming properties, including tight junctions and distinct extracellular matrix (ECM) component expression, it raises the question of whether different mechanisms might be at play^47^.

### Types of mechanical forces involved in BBB functioning

**Fig. 2:**
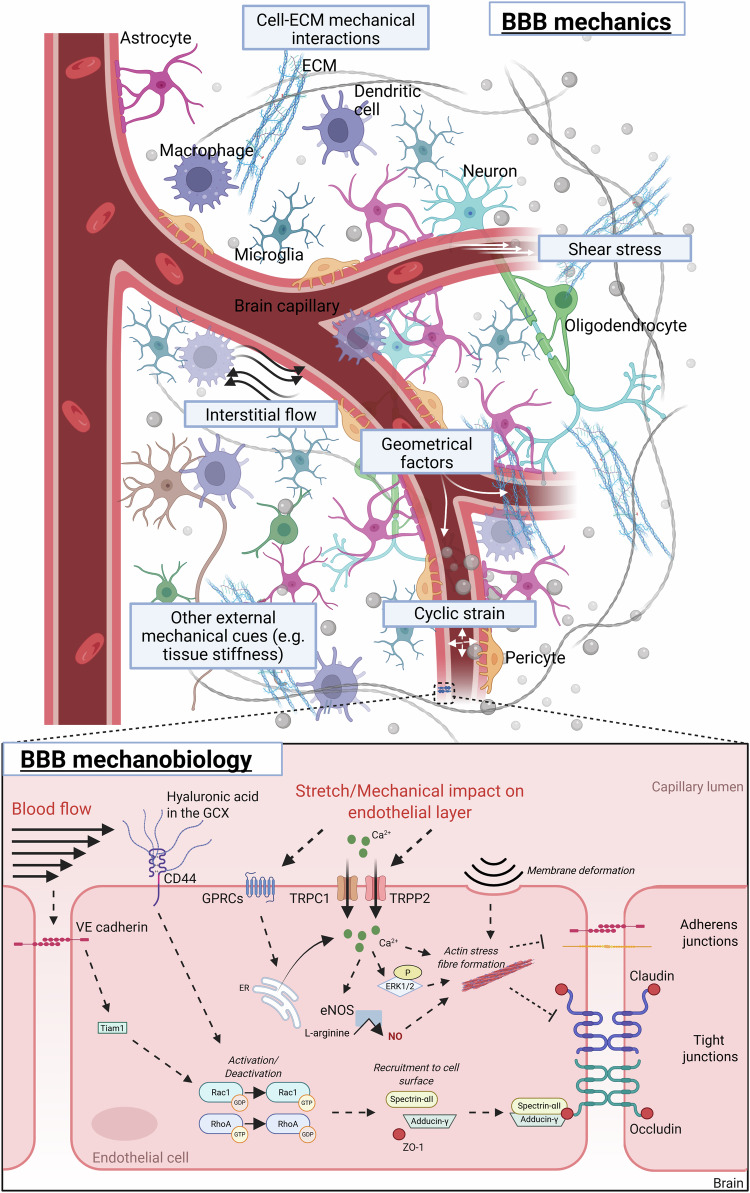
Mechanical forces at the BBB and selected mechanotransduction pathways influencing BMEC function. *Top panel*: Mechanical cues (grey boxes) acting on the BBB originate from blood flow, the mechanical properties of cells and the surrounding extracellular matrix. *Bottom panel*: Multiple mechanosensors and signalling pathways in BMECs modulate their ability to maintain a tight barrier. Shear stress is sensed by CD44 at the glycocalyx (GCX) and by VE-cadherin, which activate the RhoA/RAC1 pathway to promote junctional protein localisation at the plasma membrane. In contrast, stretch and other mechanical perturbations to the endothelium are detected by G protein–coupled receptors (GPCRs) and mechanosensitive ion channels (TRPP2 and TRPC1), which can trigger actin stress fibre formation and disrupt tight junction assembly. Further details are discussed in Section 3.2. Created in BioRender. Konig, S. (2025) https://BioRender.com/z8u1cwz.

The BBB is the main interface between the blood and the brain. It may, therefore, be exposed to mechanical cues from both the blood flow and the surrounding brain tissue. Cells and matrix components within the BBB also exhibit their own distinct mechanical characteristics. Based on this, we propose that the mechanical cues outlined in the following (summarised in Fig. 2) contribute to the regulation of BBB function.

Shear stress, generated by frictional forces acting parallel to the vessel wall, is key to normal vascular function. The shear stress experienced by the endothelium is inversely proportional to the cube of the vessel radius and directly proportional to viscosity, assuming Poiseuille flow with Newtonian fluid and steady flow conditions^48^. Direct measurement of shear stress in human brain capillaries is challenging due to the invasive nature of the required procedures. However, shear stress can be predicted from indirect measurements of cerebral blood flow, vessel diameter and blood viscosity, leading to estimates of 1–2 Pa in human brain capillaries^48^. Direct measurements via intravital imaging of rat brains have revealed highly heterogenous flow patterns with stresses ranging from 0.5 to 2.3 Pa in cerebral capillaries^49^. Several in vitro studies have previously investigated the positive influence of shear stress on BBB function by applying different shear stress levels ranging from 1.9 × 10^-4^ to 1.5 Pa^50–53^. It is important to note that when comparing the response to mechanical stimuli in different studies, one must consider the specifics of each model system. For instance, cells exposed to shear stress within a polymer microchannel may experience forces that differ significantly from those experienced by cells cultured on transwell membranes or in standard cell culture flasks due to the varying stiffness of the substrates, which can affect the cellular response. Studies have investigated the role of shear stress in different model systems, including under BMEC monoculture and co-culture conditions, and comparative flow vs. no-flow settings^39,50,54–58^. It was thus shown that physiological shear stress is positively correlated with improved vascular barrier integrity, BMEC survival, and the degree of BMEC alignment to the flow direction. In BMECs, significant changes in the gene expression of tight junction proteins and protective proteins, such as those of the ABC transporter family (including multidrug resistance transporters like P-glycoprotein) and antioxidant enzymes, result from increased shear stress^54,59,60^. ECM protein production pathways are also enriched^54,61^. Different studies have investigated the effect of shear stress on BMEC alignment. In contrast to peripheral endothelial cells, which typically align in the direction of flow, BMECs have been observed to align perpendicularly to the flow direction or maintain their cobblestone morphology under shear stress^51,58,62,63^. Only a few studies have reported BMEC alignment in the flow direction^64,65^, which may be explained by differences in flow exposure times and variations in the design of the devices used. While shear stress appears to be associated with improved barrier function, it should be noted that an abnormally high shear stress (4 Pa), which may, for example, occur during pathology, has been found to lead to a decrease in the expression of tight junction proteins as well as altered junctional morphology^66^. Besides shear stress, the intraluminal cyclic pressure within the vasculature, though highly damped at the level of the capillaries, also induces cyclic strain on the BBB. Such physiological cyclic strain was found to decrease BBB permeability and facilitate transport along the vessel wall retrograde to the applied fluid flow^67^. However, another in vitro study found a decrease in tight junction expression and morphology^66^. Besides differences in BMEC sources and co-culture methods, the contrasting results of the studies may be attributed to the use of different substrates on which BMECs were cultured, whereby either a hydrogel composed of collagen, hyaluronan and Matrigel^67^ or a simple silicone tube was used^66^. These substrates may absorb and transmit the pulsatile forces experienced by the BMECs differently, which could thus explain the discrepancy between the two studies. The distinct effects of shear stress and cyclic strain may also be highly dependent on the specific flow profiles, such as continuous, pulsatile or oscillatory. In peripheral vessels, particularly oscillatory shear (0–4 dyn/cm²) at arterial bifurcations, contributes to endothelial dysfunction and atherosclerosis. This occurs through glycocalyx disruption, cytoskeletal disorganisation^68^, downregulation of ecNOS, and upregulation of ET-1, leading to inflammation and vasoconstriction^69,70^. High shear stress (>2 Pa) counteracts these effects by suppressing VEGFA, HIF1, and ANG2, thereby maintaining vascular stability^69,71^. Differences in shear-related outcomes between the BBB and peripheral vessels may stem from unique shear magnitudes, transcriptional responses (e.g., KLF2 and FOXC2 regulating distinct targets in BBB cells)^54,68^, and pathological contexts. While peripheral atherosclerosis arises from oscillatory shear-induced ET-1/ROS signalling^69,70^, BBB dysfunction in conditions like stroke is often linked to abrupt shear loss, triggering MMP-9 and inflammatory cascades^47^. These differences highlight the importance of BBB-specific shear studies in accounting for unique transcriptional programs and barrier reinforcement mechanisms absent in peripheral models.

In addition to blood pressure and flow rate, the levels of shear stress and cyclic strain are impacted by the vessel radius and geometry and the strength of the vessel wall^72^. In addition to the vessel diameter, the curvature and bifurcations alter the blood-flow patterns and hence the mechanical forces experienced by the BMECs. In cerebral aneurysms, which occur at larger arteries rather than at the capillary level of the BBB, saccular formations appear predominantly at vessel bifurcations, curved regions and branch points since here hemodynamics are altered^73,74^. Interestingly, in Alzheimer’s disease (AD), various changes in the vessel geometry, including in microvessels, have been observed, such as vessel fragmentation, branching, and thinning, loop formation, increased vessel tortuosity, and thickening of the vascular basement membrane^72,75^. In vitro studies suggest that, in areas with disturbed flow due to bifurcations, the barrier permeability is significantly greater compared to regions with undisturbed flow^76^. Therefore, the geometrical changes observed in AD may alter the vascular flow patterns and the associated mechanical forces, which may contribute to and potentiate AD pathology through BBB breakdown. Multiscale modelling of the impact of traumatic brain injury (TBI) on BBB disruption has highlighted how vessel alignment and directionality in vessel networks determine the mechanical forces impacting the BBB (see Box 1)^77^.

Brain tissue mechanical properties also impact BBB function^1^. The brain is one of the softest tissues in the body and its stiffness changes in various neurological diseases^78–80^. Modelling studies investigating the over-pressurisation of arterioles (commonly observed during TBI) showed that the relative difference in stiffness between the arterioles and the surrounding brain tissue determines the degree of strain transmitted to the vessel surroundings^81^. For the BBB, so far, no direct correlation has been reported between brain tissue stiffness and BBB function. However, during physiological ageing as well as in AD, the brain softens, which in both cases coincides with the breakdown of the BBB^80,82^.

The macroscopically apparent brain-tissue stiffness emerges from the mechanical properties of its microstructural components, including cells, the ECM, interstitial fluid, and soluble and insoluble molecules, as well as from active or passive interactions amongst them. Because the ECM is the main determinant of brain stiffness, the ECM architecture and stiffness itself are particularly important mechanical cues influencing the BBB. Changes in the BBB matrix composition, which directly impact the ECM stiffness, have previously been observed, for example in ageing, during which changes in the number of tight junctions have also been reported^83–85^. The mechanical properties of the surrounding cells may also impact BMECs and BBB function. Under ischaemic conditions, astrocytic end-feet at the BBB reportedly undergo significant swelling, leading to temporary microvascular obstruction due to compression^86^. Pericytes, on the other hand, are highly contractile cells that, similar to smooth muscle cells, can alter capillary blood flow in response to the energy demand of the surrounding brain tissue, a process known as neurovascular coupling. Pericyte contraction induces substratum deformation and can thus directly apply a mechanical stimulus to adjacent vascular BMECs or modify the effective mechanical stiffness of the surrounding ECM^87^. Recent studies have shown that stiffer vessels with decreased vascular compliance are closely linked to pathologies characterised by barrier disruption, including cognitive decline and progression to dementia^88^. Vascular stiffening not only precedes neurodegenerative changes but also exacerbates amyloid and tau pathology, further compromising BBB integrity. Additionally, arterial stiffening and inflammation have been shown to interactively exacerbate tau pathology in older adults with mild cognitive impairment, highlighting the complex interplay between vascular mechanics and neurodegenerative processes^89^. These findings underscore the critical role of vascular compliance in maintaining BBB function and suggest that loss of compliance contributes to barrier breakdown in disease states. While in vivo and clinical studies have established strong correlations between vascular stiffness and BBB dysfunction, there remains a significant gap in the field regarding direct in vitro investigations of how matrix stiffness specifically alters tight junctions between cells with endothelial phenotypes. Most current in vitro BBB models have not systematically varied substrate stiffness to directly assess its impact on tight junction protein expression and barrier properties. Addressing this gap will be essential for elucidating the mechanistic links between mechanical cues and BBB integrity, and for developing targeted interventions to preserve barrier function in disease.

Box 1 BBB mechanics during traumatic brain injury (TBI) During TBI, strong mechanical stimuli are exerted on the brain, including the BBB, consequently showing increased permeability^184^. Animal models of TBI have demonstrated that BBB breakdown occurs both concomitantly with trauma and approximately 48 h after trauma^185,186^. During TBI, various types of stress act on the brain capillaries, including compression, tension, shear, torsion, and bending^184^. These forces inflict direct haemorrhaging of vessels but may also lead to microstructural damage of the cells and the ECM of the BBB, as similarly observed in TBI during diffuse axonal injury of neurons^184^. The vessel geometry may thus be important, as vessel branches may be more susceptible to haemorrhaging than straight segments^184^.

Intrinsic BBB mechanics may also be altered after injury, leading to BBB malfunction. In TBI, the autoregulation of cerebral blood flow often fails, leading to increased blood pressure and hence altered shear stress, cyclic strain, and interstitial flow^187^. On the other hand, increased BBB breakdown may also decrease blood flow within the brain capillaries, which may further impact tight-junction formation. Astrocyte interactions may also be disturbed since astrocytes are known to respond to mechanical impact by increasing intracellular ion levels, releasing molecules such as ATP extracellularly, and rapid swelling^188–192^. Mechanical injury of astrocytes in vitro causes the upregulation of the glial fibrillary acidic protein (GFAP), a filament protein, which coincides with cellular softening^145^. Reportedly, stiffness decreases significantly in the impacted regions immediately after injury and in the astrocytic scar that forms several days after TBI^79,193^. Such changes are likely to impact BBB mechanics and function further.

### Mechanotransduction pathways governing BBB function

Despite the increasingly recognised importance of mechanical forces on BBB function, only a few distinct BBB-linked mechanotransduction pathways have so far been identified (Fig. 2). In particular, the intraluminal glycocalyx layer (GCX) has been highlighted. It was shown that, in the peripheral vasculature, the GCX plays an important role in determining vascular permeability, although the underlying mechanisms remain open to debate^90,91^. It mediates shear-stress-induced nitric-oxide formation, which is linked to vasodilation and vascular health^47,92,93^. Interestingly, at the BBB, GCX expression is higher than in the peripheral vasculature, and GCX degradation has been suggested in pathologies such as AD, stroke and TBI as well as during ageing^94–98^. Shi et al. showed that during ageing and neurodegeneration, the GCX is dysregulated, whereby specifically mucin-domain glycoproteins are disrupted, leading to BBB dysfunction and even brain haemorrhaging in mice^98^. Notably, in the same study, the authors also showed that restoring mucin-type O-glycan biosynthesis in aged mice improved GCX structure and BBB function^98^. These findings highlight the critical role of GCX integrity in maintaining BBB function. DeOre et al. recently observed that BMECs actively probe blood flow through the cell surface receptor CD44^99^. This receptor binds to hyaluronic acid (HA) within the GCX and, in the presence of shear stress, mediates BMECs' barrier function through RhoA/RAC1 signalling^99^. The cell surface receptor syndecan, which binds to the most abundant glycosaminoglycan in the GCX, heparin sulfate, may also be implicated in the mechanotransduction^100^. Syndecan 1 has previously been shown to be involved in regulating the arrangement of the cytoskeleton in endothelial cells, even though no direct evidence currently exists for BMECs at the BBB^101^.

The well-defined junctional mechanosensory complex has also been implicated in endothelial mechanotransduction^102^. Specifically, the junctional protein VE-cadherin (involved in homotypic cell–cell adhesion) and its binding partners (platelets and endothelial cell adhesion molecule 1 (PECAM-1) and vascular endothelial cell growth factor receptor 2 (VEGF-R2)) have been shown to mediate shear-stress-induced mechanotransduction in BMECs, leading to increased RAC1 and barrier integrity via occludin^60^.

Mechanosensitive ion channels, such as the transient receptor potential (TRP), are also heavily expressed in the brain microvasculature and are involved in BBB damage in TBI^103,104^. More specifically, both TRPP2 and TRPC1 were found to modulate nitric oxide production and actin stress fibre formation during stretch-induced injury of BMECs^104^. Piezo1, another mechanosensitive cation channel, has recently gained interest for its possible role at the BBB. Piezo1 expressed at BMECs has been found to regulate pericyte proliferation during development via NOTCH signalling^105^. During cerebral infarction, knocking out Piezo1 restored tight junction protein expression by inhibiting the IL6/GPX pathway, accompanied by improved cognitive function. Pharmacological inhibition of Piezo1 during chronic cerebral hypoperfusion also improved BBB disruption and cognitive impairment, further supporting a role for Piezo1 as an important mechanosensor of capillary perfusion^106^. By applying targeted mechanical forces to a monolayer of BMECs via picosecond laser excitation to glycoprotein-targeted gold nanoparticles (AuNPs), Li et al. recently proposed that mechanical forces can activate the mechanosensitive Ca^2+^ channels TRPV4 and Piezo1 as well as mechanosensitive GPCRs. In both cases, intracellular Ca^2+^ levels are increased, resulting in the activation of the ERK1/2 phosphorylation pathway^107^. This, together with actin polymerisation due to cell membrane deformations, may then result in cytoskeletal rearrangement and contraction. Given the tight regulation of endothelial barrier integrity by the balance between actomyosin contractile forces and the tethering forces generated by cell-cell and cell–ECM interactions, such cytoskeletal contraction results in BBB opening^108^. Similarly, increased Ca^2+^ and cytoskeletal contraction were also observed when BMECs were mechanically stimulated by vertically deployed surface acoustic waves^109^.

## Blood–brain barrier mechanics during disease, development and ageing

### The potential role of mechanics in BBB development

Mechanics is a fundamental aspect of development and is crucial for healthy tissue and organ morphogenesis^110,111^. Nonetheless, much remains to be learned of its role in BBB development. During central nervous system (CNS) vascularisation, at approximately embryonic day E 11.5 of the mouse gestation, mesoderm-derived angioblasts coalesce to form the perineural vascular plexus (PNVP) along the neural tube^112,113^. Following the release of growth factors, such as VEGF, from cells in the neural tube, ECs in the PNVP undergo sprouting angiogenesis and extend towards the ventricles into the developing CNS^112–114^. By capturing signals emitted by the surrounding CNS cells (such as astrocytes, pericytes and neural cells), these sprouts develop barrier properties to form a functional BBB^115–118^.

Wingless–Int1 (Wnt) signalling in endothelial cells has been shown to play a key role in regulating angiogenesis and barrier formation, specifically in the brain but not in peripheral tissues^118,119^. More specifically, during development, astroglia, oligodendrocytes, and neurons express Wnt7a and Wnt7b, which bind to the Frizzled receptor and its coreceptors Lrp5/6, GPR124, and RECK on endothelial cells to induce canonical Wnt signalling^120^. Since Wnt7a/b signalling through GPR124/RECK is specific to the BBB, Gpr124/Reck agonists have recently been explored as potential neurovascular protective therapeutics to restore BBB function^121^. Following Wnt signalling and β-catenin stabilisation, endothelial cells acquire BBB-specific characteristics such as reduced paracellular permeability, suppressed transcytosis, induction of specific solute transporter expression, and recruitment of pericytes^115–118^. Interestingly, in addition to the well-established role of VEGF signalling in angiogenesis, Wnt signalling is also essential for brain-specific angiogenesis, ensuring that only BBB-specific endothelial cells can penetrate the developing brain^122^. Recently, it was shown that Wnt7a/b signalling specifically induces the glycosylphosphatidylinositol-anchored matrix metalloproteinase-25 (MMP25) at the tips of endothelial cells, thereby facilitating their migration by cleaving collagen IV chains^122^. Given the critical role of the Wnt pathway in brain microvasculature formation, it is not surprising that disruption of Wnt signalling during development, as shown in knockout experiments, leads to severe, brain-specific angiogenic defects and haemorrhaging^118^. Interestingly, we previously demonstrated that the loss of yes-associated protein 1 (YAP1, a known mechanotransducer) in fibroblasts during murine embryonic development also causes haemorrhaging and disrupts vascular morphogenesis in the head at E 11.5, possibly indicating a role of YAP1 in CNS vascularisation and BBB development. Importantly, YAP1 binds to the transcriptional co‑activator, β‑catenin, which is a key regulator of the above-mentioned WNT signalling pathway. YAP1 is one of the best-established factors involved in cellular mechanotransduction, where it acts as a transcriptional coactivator to translate mechanical stimuli into cellular responses^123^. Collectively, considering the importance of WNT signalling in BBB development and its linkage to YAP1, YAP1 can act as a mechanotransducer during BBB development by interacting with the WNT signalling cascade, providing regulated mechanical cues for vascular morphogenesis. Indeed, we found that upon YAP1 loss in fibroblasts, regions affected by vascular defects in the developing head of the embryo exhibited a 50% reduction in stiffness^124^. It is also well-established that stiffness patterns arise during brain development, including in the head mesoderm, which are important for instructing neuronal growth and neural crest cell migration, as well as for instructing BBB morphogenesis^111,125^.

### Distinct BBB alterations during healthy ageing may be linked to changes in BBB mechanics

It has long been established that ageing brings about changes in the brain vasculature, including the BBB, and in the surrounding brain environment^126,127^. While various age-associated pathologies, including neurodegenerative disorders, lead to a dysfunctional BBB, the BBB arguably undergoes distinctive cellular and structural alterations even during healthy ageing^126,127^. It is not yet fully understood whether the BBB becomes dysfunctional or, instead, adapts to the evolving demands of the ageing brain^126^. The concept of healthy ageing and its associated changes in the BBB have recently been extensively reviewed^126–129^. From a mechanical point of view, it can be presumed that the numerous structural and cellular changes during ageing also impact BBB mechanics, but there is only limited evidence of this at present. During healthy ageing, the peripheral vasculature stiffness increases because of changes to the ECM^130,131^. This, in turn, causes a rise in systolic blood pressure and widened pulse pressures, which affects the stress imposed on the microvasculature, including the BBB in the brain capillaries^131^, although to a lesser degree considering the lower pressure and pulsatility there. As outlined in Part 3.1, forces exhibited by blood flow (such as shear stress, cyclic strain, and interstitial flow) reportedly alter the ability of BMECs to form a functional BBB^39,50,54–58,66,67^. Thus, the increased blood-flow stress imposed on the BBB during ageing may directly impact BMEC function.

Indeed, BMECs, which also undergo senescence during ageing and in ageing-related diseases, display an increased cytoplasmic area in the ageing human white matter and a decreased number of tight junctions in aged rats^85,132–134^. In addition, various transporter proteins, including transporters important for drug delivery, were found to be altered in BMECs during ageing^135–140^. Pericytes may be lost during ageing, which may further impact the microvasculature blood flow because of their important role in neurovascular coupling^130,137^. A large body of evidence exists for age-related changes in astrocytes including an inflammatory phenotype with an increase in expression of neuroinflammatory genes, increased oxidative metabolism, and altered glutamate regulation during ageing^141–144^. It has also been reported that BBB-associated astrocytes show larger end-feet in aged rats^85^. Previous studies have revealed that astrocyte activation is associated with altered tissue mechanics^79,145^. Therefore, it is plausible that perivascular astrocytes also alter their tissue mechanics during ageing, which may impact BBB function. Recently, it was shown that a stiffening of the oligodendrocyte progenitor cell (OPC) niche during ageing causes a decrease in OPC proliferation and differentiation via the mechanoresponsive ion channel Piezo1, confirming the importance of stiffness-associated changes during ageing^146^.

Interestingly, changes in the BBB ECM are especially distinctive during ageing. In the healthy-aged human brain, the BM at the microvasculature thickens considerably^126,147^. While this could be the response to an increased pulsative blood-flow stress in the periphery, which may be transmitted to the brain capillaries, animal models of ageing that normally do not show an increased systolic blood pressure or a widened pulse pressure also display a thickened cerebral microvasculature^148^. This morphological change is accompanied by an altered BM composition, with variations in collagen, laminin, nidogen, perlecan and fibronectin, depending on the assessed brain region in humans or animals^84,148–151^. These changes may also result from an altered degradation through matrix metalloproteinases (MMPs), which are dysregulated in various vascular and neurological pathologies^80,152^. A recent study also reported the accumulation of lipid droplets and aggregates on the perivascular side at the microvasculature in aged mice, further showing a potential dysregulated mechanism of ageing at the BBB^153^. Previous in vitro studies have shown that BMECs actively respond to the surrounding ECM. For instance, altering the ECM secreted by astrocytes and pericytes directly affected barrier integrity in porcine brain capillary endothelial cells^154^. Furthermore, evidence indicates that specific binding motifs within the ECM can enhance the attachment and tight junction expression of BMECs^155^. The changes in ECM composition during ageing may alter the BBB tissue stiffness. So far, potential changes in the mechanical properties of the BBB during ageing have not yet been measured. Additionally, changes in ECM stiffness are often associated with changes in hydraulic permeability, which could impact the clearance of disease-causing substances (e.g., amyloid β, α-synuclein, phosphorylated tau) from the brain parenchyma.

### BBB mechanics as a therapeutic target

As exemplified in the section “Types of mechanical forces involved in blood–brain barrier functioning” and further discussed in Boxes 1 and 2, the mechanics of the BBB are altered across various neurological disorders. These changes may be viewed as a response to external factors, such as disrupted blood flow, increased pressure, the incorporation of stiff elements like amyloid-beta, or pathological molecular processes affecting BBB cells and the ECM. However, this perspective is overly simplistic. BBB mechanics play an active role in neurological pathologies, where mechanical alterations result from the disease and contribute to its progression. For instance, ischaemic conditions cause astrocytic end-feet to swell, leading to mechanical pressure and microvascular compression, further exacerbating brain ischaemia^86^. Such insights highlight the potential of mechanobiology-based therapies for neurological disorders affecting the BBB. These therapies could aim to either reduce external mechanical stressors or intervene with targeted strategies. For instance, various clinical investigations have focused on the relationship between arterial hypertension and dementia, including AD, with promising results for antihypertensive therapy^156^. These positive effects may at least be partly due to the restored capillary flow, thus reversing BBB pathologies, including BMEC activation, altered ECM secretion and BBB breakdown. However, such systemic interventions may not be feasible for all patients. A more targeted approach, such as blocking pericyte contraction in response to amyloid-beta while applying a vasodilator, has been shown to restore capillary flow in rodents, offering a promising strategy for reversing cerebral blood flow disturbances in AD^157^. As we further explore the mechanical changes at the BBB, mechanobiology will become increasingly important, both as a direct therapeutic target and as a key factor in assessing therapeutic efficacy.

Box 2 Linking brain tissue mechanics and BBB dysfunction in Alzheimer’s disease BBB malfunction has been observed in various neurodegenerative disorders, including at the early and advanced stages of Alzheimer’s disease (AD)^7^. Cerebral amyloid angiopathy (CAA) is a key hallmark of AD, whereby the fibrillar amyloid beta protein (Aβ), especially the beta-amyloid peptide 1–40 (Aβ1–40), accumulates in the vicinity of small vessels and capillaries, leading to BBB hyperpermeability with reduced tight junctions^10,194^. CAA has gained specific interest since it is considered a major risk factor for AD patients in developing Amyloid-related imaging abnormalities (ARIA) after undergoing anti-amyloid monoclonal antibody therapy^195^.

Amyloid plaques are stiff elements. In vivo studies have shown that astrocytes surrounding amyloid plaques upregulate mechanosensitive Piezo1 cation channels^196^. Animal models and magnetic resonance elastography measurements in humans with AD have shown that the brain softens during AD progression, coinciding with astrogliosis^82,197–199^. In regions of BBB leakage, swollen and reactive perivascular astrocytes have been observed with some evidence suggesting a decoupling of astrocytic end-feet from the endothelial cells in the BBB as well as a disruption of astrocyte-mediated tight junctions through VEGF and MMP9 signalling^200–202^. Overall, this evidence shows that BBB astrocytes respond actively to CAA, possibly by altering cellular mechanics to produce a softer phenotype. Moreover, during AD and CAA, tissue crosslinking enzymes such as lysyl oxidase or tissue transglutaminase accumulate around amyloid plaques at the vessel wall, indicating distinct mechanical changes in the vascular microenvironment^203–205^. It has also been observed that the BM thickens and undergoes significant remodelling with alterations in its composition, including decreased glycosaminoglycans^206–208^. Such changes could result from the activated microvascular endothelial cells and perivascular pericytes during AD, but they may also be a mechanical response to perturbations in capillary blood flow. In AD patients, age and cardiovascular comorbidities induce a stiffening of the central cerebral blood vessels, leading to the transmission of excessive pulsatile energy to the brain microvasculature, a phenomenon that can cause microbleeds, as often observed in patients with mild cognitive decline^72,209^. In addition, histopathological inspection of the vasculature in AD has also shown that capillaries are constricted due to Aβ deposition, leading to pericyte contraction, an effect which was halted and reversed via targeted pharmacological interventions^157^. Other geometrical alterations, including increased vessel tortuosity, loop formation, and microvessel thinning, have also been observed^210,211^. These findings suggest that vascular mechanics are an important part of CAA and blood-flow alteration in AD, and thus should be considered as a target for AD therapy.

## Emerging technologies to investigate BBB mechanobiology

Despite our increasing knowledge about the influence of mechanical cues on BBB function, much remains to be learned. For this, appropriate systems incorporated with suitable probing methods are required. Determining BBB and vascular mechanics in the living human brain would be most relevant, but is challenging as most traditional mechanical tests require contact with tissue samples or vessels for force application and probing, which may risk the health of volunteers^158^. Therefore, mechanical measurements must either be performed non-invasively or ex vivo on extracted brain samples. Various techniques have been developed to assess brain tissue mechanics. Magnetic resonance elastography (MRE), is a non-invasive method which can determine overall brain-tissue mechanics, but is limited in its inability to assess mechanical heterogeneity between different brain regions^159^. Ultrasound elastography is another commonly applied clinical technique for determining tissue elasticity, but its neurological application is limited mostly to intraoperative and neonatal use. As the skull attenuates sound waves, imaging is only possible when the brain is accessible during surgery or before the closing of the fontanelles^160,161^. Tagged magnetic resonance imaging (MRI) has also been applied to obtain quantitative strain measurements of the human brain following controlled skull motion at a sub-injury level^162^. While all of these techniques have given valuable insights, they are largely limited in their resolution and more targeted approaches suitable for the scale of the BBB are needed. As explained in earlier sections, the BBB is influenced by both the surrounding cell and brain tissue mechanics, as well as mechanical stimuli exerted by the blood flow. Thus, assessing vascular dynamics may provide important insights into mechanical alterations at the BBB during disease. Various techniques have already been clinically evaluated, including transcranial Doppler ultrasound to determine cerebral blood flow^163^, as well as different MRI techniques (DSC, DCE, ASL, IVIM) to determine specifically the flow dynamics of the brain microvasculature^164^.

Ex vivo assessment of the human brain vasculature is specifically useful for high-resolution imaging of the intact microvasculature, which cannot be achieved with current clinical techniques in the living human brain. Hierarchical phase-contrast tomography (HiP-CT) has recently been applied to image whole brains from healthy and diseased donors at the micron scale^165^. Such high-resolution imaging may provide evidence for yet unseen morphological alterations at the microvasculature, which may also indicate altered BBB mechanics. Performing direct mechanical tests ex vivo only offers limited insights, as the brain consists of highly fragile and biphasic tissue with a large quantity of water that escapes naturally or becomes trapped during brain tissue preparations^158^. In addition, the ultrasoft and highly heterogeneous mechanical properties of the brain further complicate the application of traditional mechanical measurement techniques^158^.

Animal experiments face similar challenges when applying traditional contact-dependent mechanical tests and are further limited by ethical issues^50,158^. In addition, the BBB exhibits species-specific mechanobiological differences that challenge the direct translation of animal model findings to human physiology. Structural variations, such as the complexity of tight junctions and efflux transporter expression, significantly impact barrier properties. Human BMECs form more intricate tight junction networks than rodents, influenced by the species-specific expression of claudin-5 and occludin^166,167^. While porcine models demonstrate higher transendothelial electrical resistance (TEER) than rodents, their permeability remains comparable due to larger EC size^168^. Differences in P-glycoprotein (P-gp) expression levels further complicate drug permeability assessments, as rodents exhibit higher baseline activity than humans, potentially underestimating drug toxicity in preclinical studies^168,169^. Besides differences in BMECs, cellular crosstalk and ECM interactions also differ between humans and animals. Human astrocytes secrete unique factors, such as apolipoprotein E (ApoE) and angiopoietin-1 (Ang-1), enhancing BBB tightness more effectively than rodent-derived factors^166^. Murine pericytes primarily regulate BBB stability through TGF-β signalling, whereas human pericytes engage in broader cytokine interactions, influencing immune cell trafficking^166,167^. Despite these differences, animal models remain useful for BBB mechanobiology studies, particularly with the use of a cranial window, a technique that replaces part of the skull with a synthetic optical interface^170^. This technology has been applied to measure tissue elasticity with optical coherence elastography as well as assess capillary blood flow in vivo through techniques such as two-photon microscopy or laser scanning fluorescence confocal microscopy^171–173^.

Combining advanced imaging modalities like magnetic resonance elastography with computational modelling offers a unique opportunity to decode the complex mechanics of the BBB in vivo. As explained in the section “Types of mechanical forces involved in blood–brain barrier functioning”, the mechanical forces experienced by the BBB are largely transmitted by the surrounding soft brain tissue and blood flow, thus involving complex solid/fluid mechanics phenomena. Although the complexity of the vascular network makes traditional fluid–structure interaction methods difficult, poroelasticity-based approaches have recently been successful. For instance, Vardakis et al. developed a patient-specific three-dimensional multiple-network poroelastic theory-based model to investigate the spatio-temporal transport of fluid between the vasculature system, the cerebrospinal and interstitial fluid, and the brain parenchyma^174^. By applying this model to both patients with mild cognitive decline and healthy individuals, the authors could predict the swelling of the neurovascular unit during disease.

**Fig. 3:**
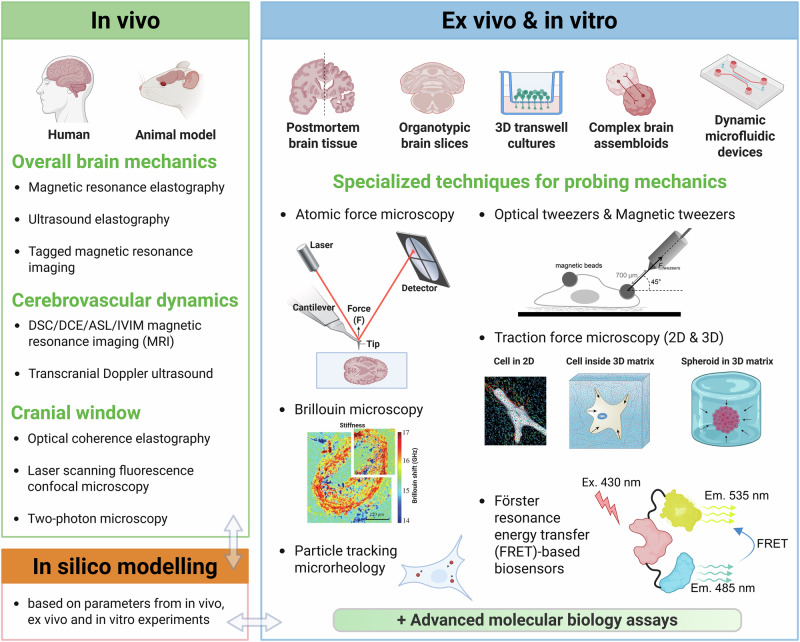
Technologies to study BBB mechanics. A range of experimental approaches used to investigate mechanical properties and mechanobiology at the BBB and in the surrounding brain, spanning in vivo, ex vivo, and in vitro contexts. In vivo methods enable assessment of whole-brain and cerebrovascular mechanics, as well as high-resolution imaging through cranial windows. In vitro and ex vivo techniques, detailed in Box 3, allow controlled mechanical measurements and manipulation at the cellular and tissue levels. These complementary strategies, combined with advanced molecular techniques and computational modelling, provide a comprehensive framework for studying biomechanics and mechanotransduction across multiple biological scales. For further details on the specific techniques, see Box 3. Elements of this figure are taken from the following references^179–183^. Adapted from refs. ^179,180,182^, and reproduced from refs. ^181,183^. Created in BioRender. Konig, S. (2025) https://BioRender.com/683mj4q.

In vitro BBB models are low-cost and increasingly robust, offering a controlled environment that can be inspected at a high spatial and temporal resolution with various mechanical measurement techniques. Some commonly used experimental techniques that may be useful to study BBB tissue mechanics in vitro are summarised in Box 3 and are further explored in another review^36^. Combining such probing techniques with suitable in vitro models may provide important novel insights. BBB in vitro models have significantly developed over the recent years, with advances in three-dimensional (3D) cell culture, microfluidic systems, and human iPSC technology enabling the creation of complex multicellular models that mimic human BBB physiology and architecture with high fidelity^50,175,176^. The development and choice of in vitro models for BBB mechanical studies depend critically on the specific study aims. For example, either a 2D or a 3D in vitro BBB model can be used, depending on the chosen probing technique and its requirements for connecting to the model. However, while the ease of assessment is important, relevant insights into BBB mechanics can only be gained by appropriately balancing model simplicity against biomimicry. Figure 3 shows different examples of how BBB mechanics can be investigated and combined with suitable probing methods.

Box 3 Common techniques for probing cellular and tissue mechanics in vitro Researchers probe cell mechanics either by applying controlled forces or deformations to cells and their extracellular environment and measuring the responses, or alternatively by probing the ability of cells themselves to generate forces and deform their environment. Used concomitantly with the mechanical measurements, imaging and molecular biology techniques can be utilised to measure cellular morphological and biological changes and elucidate mechanotransduction pathways. (Fig. 3 shows schematics of the discussed techniques).

Techniques applying controlled forces and deformations:

*Atomic force microscopy (AFM)*: AFM, a surface-characterisation technique, involves applying and monitoring forces and deformations at a high spatiotemporal resolution and at scales suitable for soft cellular materials. AFM experiments involve pressing a micron-sized tip (attached to an AFM cantilever) against a sample while measuring the resulting deformations (indentations). By using an appropriate contact model, the sample stiffness can then be determined from the measured force versus indentation distance relationship^212^.

*Particle tracking microrheology (PTM)*: In contrast to techniques that focus on the mechanical properties of cells or whole tissue, PTM allows localised mechanical measurements within the cell cytoplasm. This technique relies on trapping particles inside the cell and measuring their thermal fluctuations under the influence of local elastic and viscous resistances^213^.

*Optical tweezers*: Optical-tweezer experiments use a laser beam to stably trap small dielectric microparticles. This effect, arising from the momentum imparted by the laser beam as it refracts at a refractive-index interface, allows the manipulation of small particles incorporated within a cell or bound to the cell membrane to assess various intracellular or extracellular forces^214^.

*Magnetic tweezers/magnetic bead cytometry*: This technique applies controlled magnetic forces on magnetic beads incorporated within a biological sample. The beads are either bound to biological entities or incorporated into tissue models to perform rheology measurements^215^.

*Brillouin microscopy*: Brillouin microscopy measures the local mechanical properties of a sample by analysing the frequency shift of laser light scattered by acoustic phonons. This technique enables quantitative, contact-free mechanical mapping with high spatial resolution. Operating label-free and under physiological conditions, it provides 3D mechanical images of cells and tissues without disrupting the sample^216,217^.

Techniques for monitoring forces and deformations generated by cells and the surrounding environment:

*Traction force microscopy (TFM)*: Cells apply traction forces on their surroundings to spread and migrate. TFM quantifies these forces by monitoring the displacement of a compliant substrate as cells deform it. The cellular force exerted per unit area can then be calculated from the substrate displacement. Fluorescent beads incorporated within a hydrogel or micropillar assays are commonly used for monitoring these deformations^218^.

*Förster or fluorescence resonance energy transfer (FRET)-based biosensors*: During FRET, non-radiative energy is transferred via dipole–dipole coupling from an excited donor fluorophore to an acceptor fluorophore that emits fluorescence at a different wavelength than the donor. Since the amount of energy transferred, and hence the resulting emittance, depends on the acceptor–donor distance, FRET-based biosensors are constructed by incorporating fluorophores linked by an elastic spacer into mechanotransduction proteins. Depending on the FRET efficiency, the tension exerted across the protein and the biosensor can be determined^219^.

## Conclusions and future directions

In summary, the past few years have increasingly highlighted the importance of mechanical factors in BBB function. Initial studies have now also begun to uncover their role in BBB pathology. While much of the focus has been on blood-flow-mediated forces, the intrinsic mechanical properties of the BBB and the surrounding tissues, as well as the forces generated by cells and transmitted via the ECM, remain to be fully identified and explored.

Several questions are of fundamental importance and should serve as the focus for future investigations:

1. To what degree are the mechanical cues mentioned in the section “Types of mechanical forces involved in blood–brain barrier functioning” essential for healthy BBB function?
2. What further underlying BBB mechanotransduction pathways exist other than the ones mentioned in the section “Mechanotransduction pathways governing BBB function”?
3. Following the evidence given in the section “Blood–brain barrier mechanics during disease, development and ageing”, it is essential that we decipher how mechanical cues and their underlying mechanotransduction pathways change at the BBB in neurological disorders and ageing. Could these be targeted as part of a more comprehensive therapeutic strategy?

To comprehensively explore these topics, future research should integrate recent technological advancements in vascular and brain mechanics assessment with sophisticated biomimetic BBB in vitro models while also prioritising the development of high-resolution, non-invasive tools capable of real-time monitoring of mechanical changes in the BBB under pathological conditions. Unravelling the role of BBB mechanics holds significant promise, as nearly all neurological disorders are associated with vascular dysfunction. A deeper understanding of BBB mechanobiology could provide a transformative framework for redefining neurovascular health and uncovering innovative therapeutic strategies.

### Reporting summary

Further information on research design is available in the Nature Portfolio Reporting Summary linked to this article.

## Supplementary information

Reporting Summary
